# The complete chloroplast genome and phylogenetic analysis of *Oxytropis kansuensis* Bunge (Fabaceae)

**DOI:** 10.1080/23802359.2025.2519213

**Published:** 2025-06-17

**Authors:** Dan Lei, Hai-Tao Ma, Qi-Yin Chen, Bei Jiang, Yong-Zeng Zhang

**Affiliations:** a Yunnan Key Laboratory of Screening and Research on Anti-pathogenic Plant Resources from Western Yunnan, Dali, China; b College of Pharmacy & Institute of Materia Medica, Dali University, Dali, China

**Keywords:** *Oxytropis kansuensis*, Fabaceae, complete chloroplast genome, phylogenetic analysis

## Abstract

*Oxytropis kansuensis* Bunge holds significant medicinal and ecological values. Nevertheless, the absence of a complete chloroplast genome has impeded our ability to better understand and utilize this plant. In this study, the complete chloroplast genome of *O. kansuensis* was sequenced and *de novo* assembled for the first time. The total genome length was determined to be 127,115 bp, with a guanine–cytosine (GC) content of 34.29%. It was observed that the inverted repeat (IR) region was absent in this species. A total of 110 genes were annotated, including 76 PCGs, 30 tRNA genes, and four rRNA genes. A phylogenetic tree was constructed from the complete chloroplast genome sequences of 10 *Oxytropis* species and seven *Astragalus* species. The result indicated that *Oxytropis* and *Astragalus* clearly form distinct monophyletic lineages, respectively, and *O. kansuensis* is most closely related to the *O. ochrocephala*. This research not only enriches the genomic database but also establishes a firm foundation for future studies on *O. kansuensis*. Furthermore, it offers compelling scientific evidence to advance broader phylogenetic and evolutionary investigations of *Oxytropis*.

## Introduction

*Oxytropis* DC. (Fabaceae: Papilionoideae) is perennial stemmed or stemless herbs. While these plants closely resemble *Astragalus*, they can be distinguished by the presence of a beak at the apex of the keel petal (Zhu et al. 2010). There are more than 310 species in the world, predominantly distributed across the northern hemisphere, and 133 species were found in China, primarily occurring in Gansu, Qinghai, Sichuan, Yunnan, and Tibet (Zhu et al. 2010). Despite its inherent toxicity, species of *Oxytropis* possess medicinal and ecological values, for example, *O. kansuensis* Bunge is one of the poisonous plant species, but also has hemostatic, analgesic, detoxifying, healing, and anti-inflammatory properties (Li et al. 2018). Additionally, with its well-developed root system, *O. kansuensis* effectively stabilizes soil and prevents sand erosion. These traits make it highly suitable for ecological restoration, alpine soil and water conservation, and the rehabilitation of degraded grasslands (Zhu et al. 2010).

At present, the studies on *Oxytropis kansuensis* were mainly focused on chemical composition (Gong 2010), pharmacological activity (Liu et al. 2008; Wang et al. 2009), and endophytes associations (Zhao et al. 2009). However, the structural characteristics of the complete chloroplast genome of *O. kansuensis* have not yet been reported, and the intraspecific relationships within *Oxytropis* still remain unclear. To ascertain the phylogenetic position of *O. kansuensis*, facilitate a comprehensive understanding of its phylogenetic relationships with closely related species, and elucidate its evolutionary history, the present study successfully assembled and annotated the chloroplast genes of *O. kansuensis*. Furthermore, a phylogenetic tree was constructed using the chloroplast genes of 18 additional species available in the NCBI database. This study provides valuable scientific evidence for further studies on *O. kansuensis* and the phylogeny of *Oxytropis*, laying a solid foundation for future investigations in this field.

## Materials and methods

**Figure 1.**
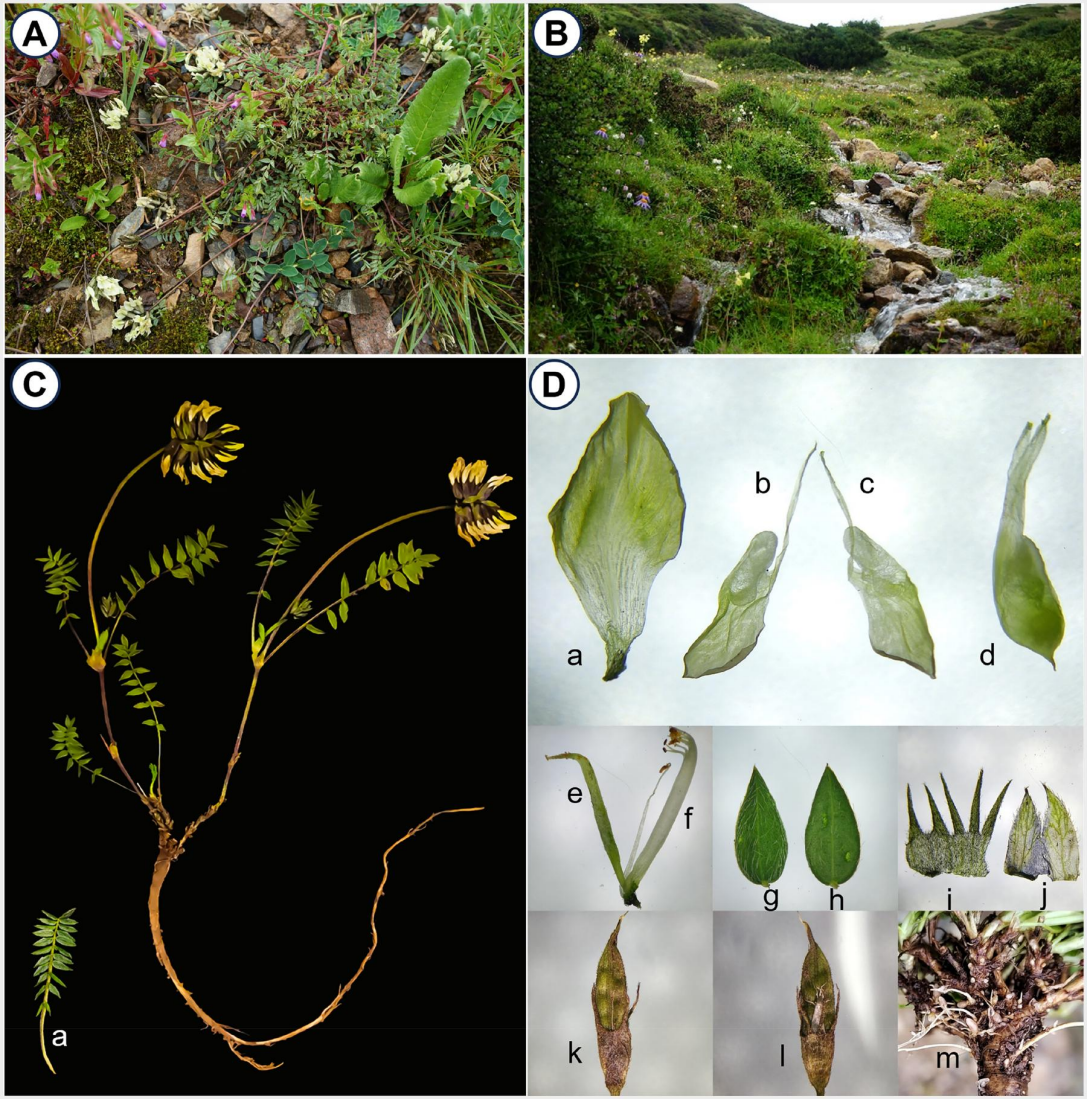
*O. kansuensis* (photographed by Dan Lei at Baima Mountain, Yunnan Province, China). (A) Habit; (B) habitat ; (C) individual (show a complete leaf sequence), (D) detail: a, standard; b and c, wings; d, keel; e, pistils; f, stamen; g, adaxial leaflet surface; h, abaxial leaflet surface; i, calyx (unfold); j, bracts (unfold); k and l, legumes; m, root nodule.

*Oxytropis kansuensis* (Figure 1) was collected on 24 July 2023 from Baima Mountain, Deqin County, Diqing Tibetan Autonomous Prefecture, Yunnan Province, China (28°20′10.896″N, 99°4′58.332″E). The leaves were desiccated using silica gel for subsequent analyses. We conducted an in-depth observation and anatomical study on the living specimens using a biomicroscope (Jiangnan JSZ5). We found that the leaflets numbered between 17 and 23. The abaxial surfaces were sparsely covered with white appressed pubescence, while the adaxial surfaces were densely so. The keel tip beak was short-triangular in shape. The ovary was covered with black pubescence. The pods were membranous and densely covered with appressed black pubescence (Figure 1(D)). All these features were consistent with the morphological characteristics of *O. kansuensis* as described in the Flora of China (Zhu et al. 2010). The voucher specimen (No. 20230724-1; contact: Zhang Yongzeng, cell_zyz@163.com) has been deposited in the Yunnan key Laboratory of Screening and Research on Anti-Pathogenic Plant Resources from Western Yunnan.

Chloroplast genomic DNA was extracted using a modified CTAB method (Doyle 1987), and the quality and concentration of the DNA were assessed using 0.7% agarose gel electrophoresis and spectrophotometry (Bio-Rad, Hercules, CA). The DNA was sheared to obtain fragments of approximately 350 bp for library construction. Subsequently, the DNA libraries were subjected to sequencing on the DNBSEQ-T7 sequencing platform. After sequencing, low quality sequences were removed using fastp v.0.23.2 software (Chen 2023). The sequencing coverage depth was subsequently determined using Samtools software (Li et al. 2009). The entire sequencing process was conducted by Wuhan Benagen Co. (Wuhan, China).

We performed the *de novo* assembly of the complete chloroplast genome of *O. kansuensis* using GetOrganelle v1.7.5 (Jin et al. 2020), annotated the chloroplast genome with CPGAVAS2 (Shi et al. 2019), visualized the chloroplast genome map using OGDRAW software (Greiner et al. 2019), and each annotation error in the chloroplast genome was corrected using CPGView software (Liu et al. 2023) and Apollo software (Lewis et al. 2002) to manually modify and refine the annotations. Finally, the annotated genome sequences were submitted to GenBank under the accession number PQ790176.1.

**Figure 2.**
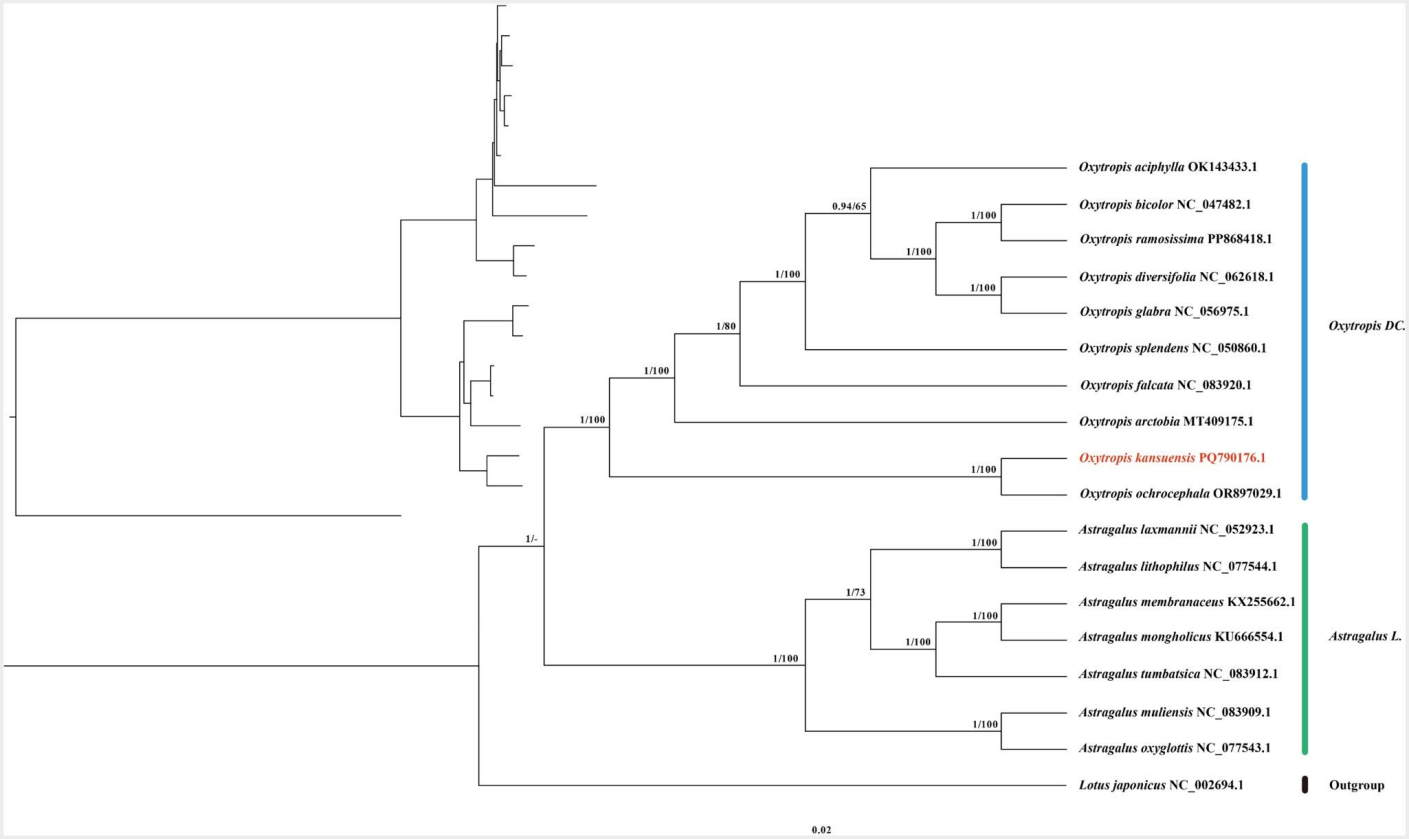
Phylogenetic tree of species of the genera *Oxytropis* and *Astragalus* based on complete chloroplast genome sequences. *Lotus japonicus* is the outgroup, and *O. kansuensis* is shown in red, with nodes labeled with ML bootstrap support values (BS) and Bayesian posterior probabilities (PPs), separated by slashes. The species sequence is as follows: *O. kansuensis* (PQ790176.1), *O. bicolor* (NC_047482.1) (Su et al. 2019), *O. ramosissima* (PP868418.1), *O. diversifolia* (NC_062618.1), *O. glabra* (NC_056975.1) (Liu et al. 2021), *O. aciphylla* (OK143433.1), *O. splendens* (NC_050860.1), *O. arctobia* (MT409175.1), *O. falcata* (NC_083920.1), *O. ochrocephala* (OR897029.1) (Hu et al. 2024), *A. laxmannii* (NC_052923.1), *A. lithophilus* (NC_077544.1), *A. membranaceus* (KX255662.1) (Wang et al. 2016), *A. mongholicus* (KU666554.1) (Lei et al. 2016), *A. tumbatsica* (NC_083912.1), *A. muliensis* (NC_083909.1), *A. oxyglottis* (NC_077543.1), and *L. japonicus* (NC_002694.1) (Kato et al. 2000).

The complete chloroplast genome sequences of 10 *Oxytropis* species and seven *Astragalus* species were downloaded from the NCBI database (all accession numbers were exhibited in the legend of Figure 2). *Lotus japonicus* (Regel) K. Larsen (accession number: NC_002694.1) was used as outgroup. The alignment of all genomic sequences was conducted using MAFFT v7 implemented in Geneious Prime (Katoh et al. 2002). Best-fit models for nucleotide substitutions were determined for both maximum-likelihood (ML) and Bayesian inference (BI) analyses using Phylosuite v1.2.3 software (Zhang et al. 2020), which selects the best-fit model for nucleotide substitutions based on the Akaike information criterion (AIC) to ensure suitability for downstream analyses. In the BI analysis, the sequences were sampled for 1 million generations of Markov Chain Monte Carlo (MCMC) using the GTR + G + F model, with one tree sampled every 1000 generations, and first 25% excluded as burn-in. In the ML analysis, 10,000 bootstrap repetitions were performed using the TIM + F + I + I + R5 model. The final visualization was performed using FigTree v1.4.4 (Rambaut 2018).

## Results

**Figure 3.**
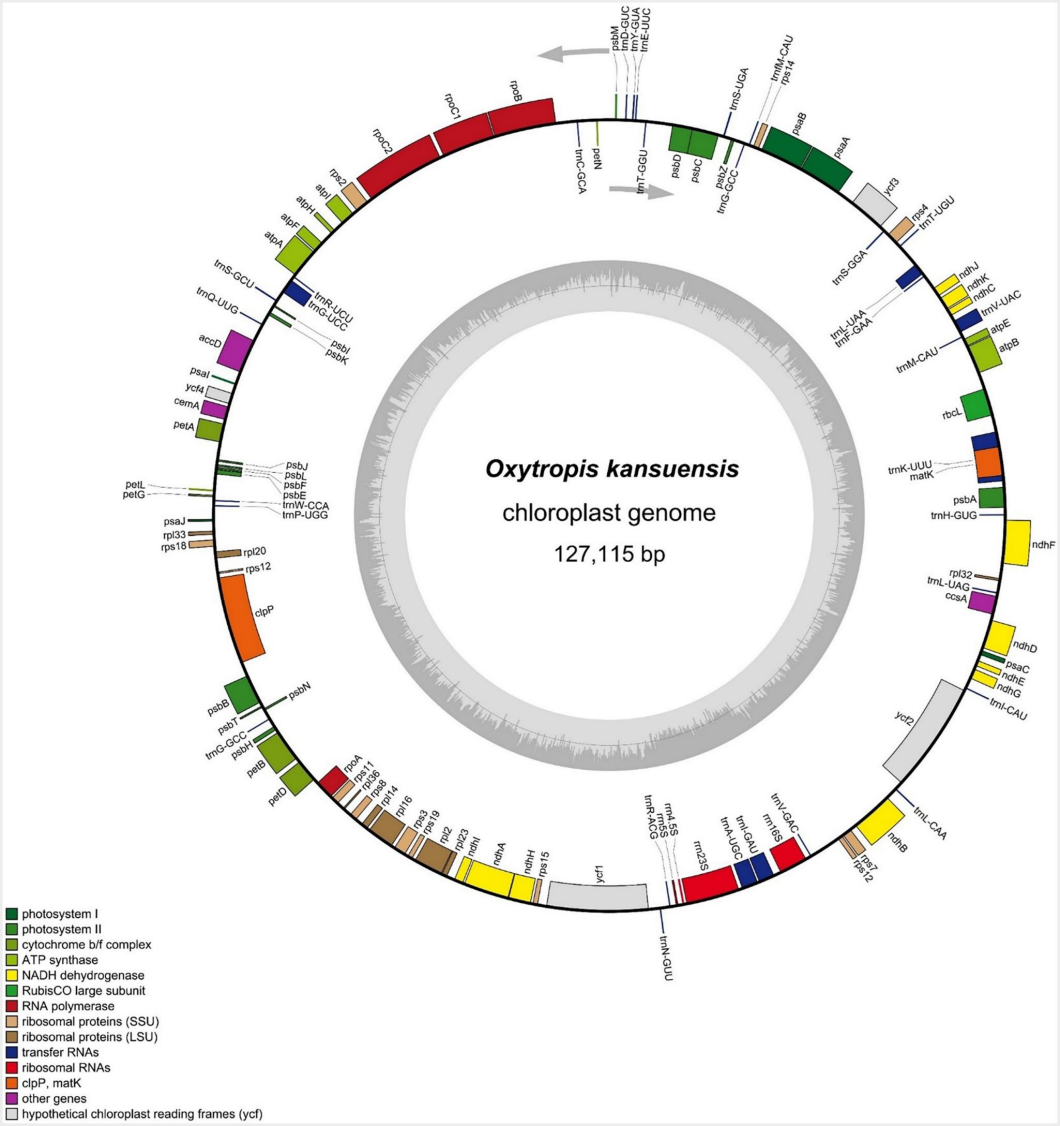
Gene map of the cp genome of *O. kansuensis.* Genes plotted inside the circles are transcribed clockwise and genes plotted outside the circles are transcribed counterclockwise. The inner dark grey circle corresponds to GC content and the inner light grey circle corresponds to AT content. Different colors represent different genes in different functional groups.

The results demonstrated that the primary structure of the *O. kansuensis* chloroplast genome was a single circular molecule, with a total length of 127,115 base pairs (bp) and an average guanine–cytosine (GC) content of 34.29% (Figure 3**)**. The depth of coverage is illustrated in Figure S1. The genome contains a total of 110 genes, including 76 protein-coding genes (PCGs), 30 transfer RNA (tRNA) genes, and four ribosomal RNA (rRNA) genes (Table S1). Our results show that the structural inverted repeat (IR) region of *O. kansuensis* was absent. Furthermore, analysis of the coding-sequence genes of *O. kansuensis* revealed the presence of nine genes that feature cis-splicing, namely *rpo*C1, *clp*P, *pet*B, *pet*D, *rpl*16, *rpl*2, *ndh*A, *ndh*B, and *ycf*3. Among these, eight genes (*rpo*C1, *clp*P, *pet*B, *pet*D, *rpl*16, *rpl*2, *ndh*A, and *ndh*B) were found to contain a single intron, whereas the *ycf*3 gene exhibited two introns, as illustrated in Figure S2. Furthermore, 111 types of simple sequence repeats (SSRs) were found, including monomeric (59), dimeric (30), trimeric (12), tetrameric (7), and pentameric (3) types, as well as long sequence repeats (LSRs) containing 370 forward repeats, 77 tandem repeats, one reverse repeat, and 25 palindromic repeats (Figure S3).

The phylogenetic tree topologies for the BI and ML analyses are essentially identical; therefore, only the BI tree is shown, displaying the posterior probabilities (PPs) form the BI analysis and the bootstrap values form the ML analysis (Figure 2). As demonstrated in the phylogenetic tree, both *Oxytropis* and *Astragalus* species exhibited well-defined clustering patterns, with *O. kansuensis*, the focus of this study, demonstrating the closest relationship to *O. ochrocephala* Bunge.

## Discussion and conclusions

As an essential component of plant cells, the chloroplast genome holds significant value in various scientific disciplines (Ran et al. 2024; Zhang et al. 2024). It plays a crucial role in species identification and the exploration of evolutionary relationships. The chloroplast genome of angiosperms is characterized by its high degree of conservation and typical tetrameric structure (Xu et al. 2023), with the IR region typically containing vital genes that play a pivotal role in gene expression. However, prior research has shown that the inverted repeat-lacking clade (IRLC), which included several Fabaceae species, exhibits a commonality in the partial deletion of the IR region (Su et al. 2021). It is worth noting that, in this study, the structural IR region of *O. kansuensis* is absent. This leads us to ponder whether it is one of the species characterized by a complex chloroplast genome evolution. Such structural variation holds the potential to influence gene expression and, furthermore, to impact species diversity and evolution (Li et al. 2021). Even more, deletions in the IR region have been observed in other species of *Oxytropis*, like *O. ochrocephala*, *O. bicolor*, *O. glabra*, and *O. aciphylla* (Su et al. 2019; Liu et al. 2021; Bei et al. 2022; Hu et al. 2024), and this chloroplast genome characteristic clearly differs from the typical tetrad structure of angiosperms (Xu et al. 2023). Therefore, we propose that the deletion of the IR region may be a common feature of *Oxytropis* and serve as one of the distinctive indicators reflecting the evolutionary dynamics within this taxonomic group.

Phylogenetic analyses of chloroplast genome sequences indicate that *Oxytropis kansuensis* is closely related to *O. ochrocephala* with strong statistical support (ML-BS = 100, BI-PP = 1; see Figure 2). However, the phylogenetic tree was constructed based on a limited number of species, potentially failing to fully capture the intricate interspecific relationships within *Oxytropis*. This constraint may introduce gaps in our understanding, as the diversity and evolutionary nuances among species could be underrepresented. Therefore, it is imperative to expand the dataset to encompass a broader taxonomic range in future research. The present study offers comprehensive information of the chloroplast genome of *O. kansuensis* and enriching the existing database of chloroplast genomes within *Oxytropis*. This research not only expands the genomic database but also lays a solid foundation for future investigations into *O. kansuensis* and provides robust scientific evidence to support broader phylogenetic and evolutionary studies of *Oxytropis*.

## Supplementary Material

Supplementary Data.docx

## Data Availability

The complete chloroplast genome sequence of *O. kansuensis* in this study has been submitted to the NCBI database under the accession number PQ790176.1. The associated BioProject, BioSample, and SRA numbers are PRJNA1199728, SAMN45889030, and SRR31773688, respectively.

## References

[CIT0001] Bei ZL, Zhang L, Tian XJ. 2022. Characterization of the complete chloroplast genome of *Oxytropis aciphylla* Ledeb. (Leguminosae). Mitochondrial DNA B Resour. 7(9):1756–1757. doi:10.1080/23802359.2022.2124822. 36213863 PMC9542791

[CIT0002] Chen S. 2023. Ultrafast one-pass FASTQ data preprocessing, quality control, and deduplication using fastp. Imeta. 2(2):e107. doi:10.1002/imt2.107. 38868435 PMC10989850

[CIT0003] Doyle J. 1987. A rapid DNA isolation procedure for small quantities of fresh leaf tissue. Phytochem Bull. 19(1):11–15.

[CIT0004] Gong HF. 2010. Studies on chemical constituents of *Oxytropis kansuensis* Bunge. Lanzhou: Lanzhou University of Technology.

[CIT0005] Greiner S, Lehwark P, Bock R. 2019. OrganellarGenomeDRAW (OGDRAW) version 1.3.1: expanded toolkit for the graphical visualization of organellar genomes. Nucleic Acids Res. 47(W1):W59–W64. doi:10.1093/nar/gkz238. 30949694 PMC6602502

[CIT0006] Hu X, Liu Y, Chen J, Su X. 2024. The complete chloroplast genome of *Oxytropis ochrocephala* Bunge 1874 (Fabaceae) and its phylogenetic analysis. Mitochondrial DNA B Resour. 9(5):641–646. doi:10.1080/23802359.2024.2350626. 38770145 PMC11104710

[CIT0007] Jin JJ, Yu WB, Yang JB, Song Y, dePamphilis CW, Yi TS, Li DZ. 2020. GetOrganelle: a fast and versatile toolkit for accurate de novo assembly of organelle genomes. Genome Biol. 21(1):241. doi:10.1186/s13059-020-02154-5. 32912315 PMC7488116

[CIT0008] Kato T, Kaneko T, Sato S, Nakamura Y, Tabata S. 2000. Complete structure of the chloroplast genome of a legume, *Lotus japonicus*. DNA Res. 7(6):323–330. doi:10.1093/dnares/7.6.323. 11214967

[CIT0009] Katoh K, Misawa K, Kuma K, Miyata T. 2002. MAFFT: a novel method for rapid multiple sequence alignment based on fast Fourier transform. Nucleic Acids Res. 30(14):3059–3066. doi:10.1093/nar/gkf436. 12136088 PMC135756

[CIT0010] Lei W, Ni D, Wang Y, Shao J, Wang X, Yang D, Wang J, Chen H, Liu C. 2016. Intraspecific and heteroplasmic variations, gene losses and inversions in the chloroplast genome of *Astragalus membranaceus*. Sci Rep. 6(1):21669. doi:10.1038/srep21669. 26899134 PMC4761949

[CIT0011] Lewis SE, Searle SMJ, Harris N, Gibson M, Lyer V, Richter J, Wiel C, Bayraktaroglu L, Birney E, Crosby MA, et al. 2002. Apollo: a sequence annotation editor. Genome Biol. 3(12):RESEARCH0082. doi:10.1186/gb-2002-3-12-research0082. 12537571 PMC151184

[CIT0012] Li H, Handsaker B, Wysoker A, Fennell T, Ruan J, Homer N, Marth G, Abecasis G, Durbin R, 1000 Genome Project Data Processing Subgroup. 2009. The Sequence Alignment/Map format and SAMtools. Bioinformatics. 25(16):2078–2079. doi:10.1093/bioinformatics/btp352. 19505943 PMC2723002

[CIT0013] Li HY, Shen DD, Huang XY, Ge B, Meng M. 2018. Research progress of *Oxytropis kansuensis.* Chin Tradit Patent Med. 40(3):663–670.

[CIT0014] Li J, Tang J, Zeng S, Han F, Yuan J, Yu J. 2021. Comparative plastid genomics of four *Pilea* (Urticaceae) species: insight into interspecific plastid genome diversity in Pilea. BMC Plant Biol. 21(1):25. doi:10.1186/s12870-020-02793-7. 33413130 PMC7792329

[CIT0015] Liu M, Wang MJ, Liu W, Yang AJ, Wang CY, Liu JX, Li M, Di DL. 2008. Study on anti-tumor effects and immunity function of *Oxytropis kansuensis* alkaloid fraction. Mod Med J China. 10(12):8–11.

[CIT0016] Liu S, Li YR, Si W, Qu WR, Yang TG, Wu ZH, Jiao PP. 2021. Complete chloroplast genome sequence of *Oxytropis glabra* (Leguminosae). Mitochondrial DNA B Resour. 6(9):2478–2479. doi:10.1080/23802359.2021.1914228. 34368449 PMC8317949

[CIT0017] Liu SY, Ni Y, Li JL, Zhang XY, Yang HY, Chen HM, Liu C. 2023. CPGView: a package for visualizing detailed chloroplast genome structures. Mol Ecol Resour. 23(3):694–704. doi:10.1111/1755-0998.13729. 36587992

[CIT0018] Rambaut A. 2018. FigTree v 1.4.4. Edinburgh: University of Edinburgh.

[CIT0019] Ran ZH, Li Z, Xiao X, An MT, Yan C. 2024. Complete chloroplast genomes of 13 species of sect. *Tuberculata Chang* (*Camellia* L.): genomic features, comparative analysis, and phylogenetic relationships. BMC Genomics. 25(1):108. 38267876 10.1186/s12864-024-09982-w PMC10809650

[CIT0020] Shi LC, Chen HM, Jiang M, Wang LQ, Wu X, Huang LF, Liu C. 2019. CPGAVAS2, an integrated plastome sequence annotator and analyzer. Nucleic Acids Res. 47(W1):W65–W73. doi:10.1093/nar/gkz345. 31066451 PMC6602467

[CIT0021] Su C, Duan L, Liu PL, Liu J, Chang ZY, Wen J. 2021. Chloroplast phylogenomics and character evolution of eastern Asian *Astragalus* (Leguminosae): tackling the phylogenetic structure of the largest genus of flowering plants in Asia. Mol Phylogenet Evol. 156:107025. doi:10.1016/j.ympev.2020.107025. 33271371

[CIT0022] Su C, Liu PL, Chang ZY, Wen J. 2019. The complete chloroplast genome sequence of *Oxytropis bicolor* Bunge (Fabaceae). Mitochondrial DNA B Resour. 4(2):3762–3763. doi:10.1080/23802359.2019.1682479. 33366179 PMC7707422

[CIT0023] Wang B, Chen H, Ma H, Zhang H, Lei W, Wu W, Shao J, Jiang M, Zhang H, Jia Z, et al. 2016. Complete plastid genome of *Astragalus membranaceus* (Fisch.) Bunge var. *membranaceus*. Mitochondrial DNA B Resour. 1(1):517–519. doi:10.1080/23802359.2016.1197057. 33473540 PMC7800798

[CIT0024] Wang MJ, Liu M, Liu W, Yang AJ, Wang CY, Liu JX, Li M, Di DL. 2009. In vivo anti-tumor effect of *Oxytropis kansuensis* alkaloid fraction and its influence on immune function. Chin J Integr Tradit West Med. 29(11):1009–1011. 20329613

[CIT0025] Xu S, Teng K, Zhang H, Gao K, Wu J, Duan L, Yue Y, Fan X. 2023. Chloroplast genomes of four *Carex* species: long repetitive sequences trigger dramatic changes in chloroplast genome structure. Front Plant Sci. 14:1100876. doi:10.3389/fpls.2023.1100876. 36778700 PMC9911286

[CIT0026] Zhang D, Gao F, Jakovlić I, Zou H, Zhang J, Li WX, Wang GT. 2020. PhyloSuite: an integrated and scalable desktop platform for streamlined molecular sequence data management and evolutionary phylogenetics studies. Mol Ecol Resour. 20(1):348–355. doi:10.1111/1755-0998.13096. 31599058

[CIT0027] Zhang N, Huang K, Xie P, Deng A, Tang X, Jiang M, Mo P, Yin H, Huang R, Liang J, et al. 2024. Chloroplast genome analysis and evolutionary insights in the versatile medicinal plant *Calendula officinalis* L. Sci Rep. 14(1):9662. doi:10.1038/s41598-024-60455-2. 38671173 PMC11053094

[CIT0028] Zhao XH, He X, Wang JN, Song YM, Geng GX, Wang GH. 2009. Biodegradation of swainsonine by *Acinetobacter calcoaceticus* strain YLZZ-1 and its isolation and identification. Biodegradation. 20(3):331–338. doi:10.1007/s10532-008-9224-0. 18931977

[CIT0029] Zhu XY, Welsh SL, Ohashi H. 2010. *Oxytropis*. In: Wu ZY, Raven P, editor. Flora of China. Vol. 10; p. 453–500. https://www.iplant.cn/info/Oxytropis.

