# Supplementary material for: The complete chloroplast genome and phylogenetic analysis of Oxytropis kansuensis Bunge (Fabaceae)
Source: Mitochondrial DNA B Resour. 2025 Jun 17;10(7):584–9. doi: 10.1080/23802359.2025.2519213 (PMC12175192; doi:10.1080/23802359.2025.2519213)
Supplement: Supplementary Data.docx [file TMDN_A_2519213_SM5691.docx]

**Figure S1.** The map of Sequencing Depth and Coverage.The x-axis shows the genomic position (in base pairs, bp), and the y-axis shows the sequencing depth.

**
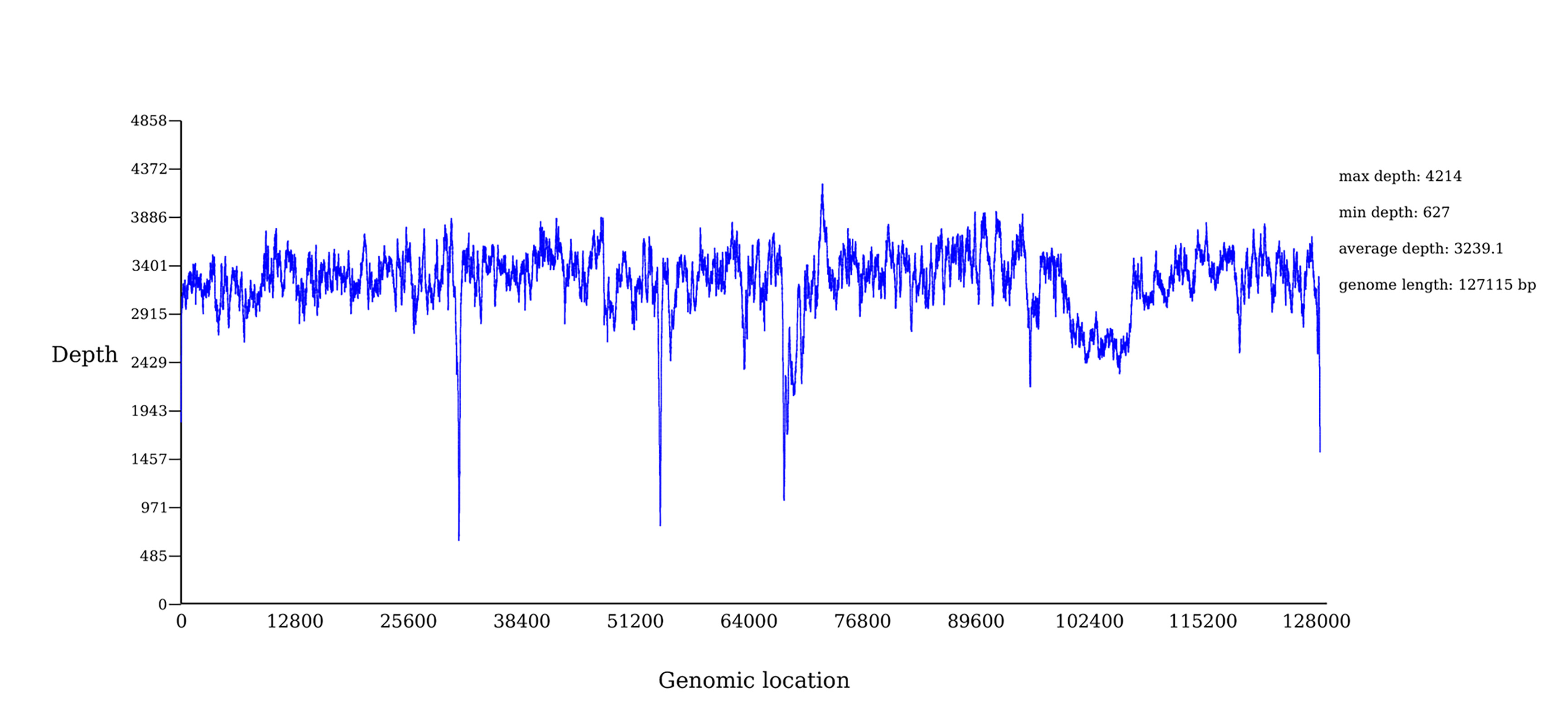
**

**Figure S2.** The map of cis-splicing genes in the chloroplast genome of *Oxytropis kansuensis*. The gene names are shown on the left, and the gene structures are on the right.


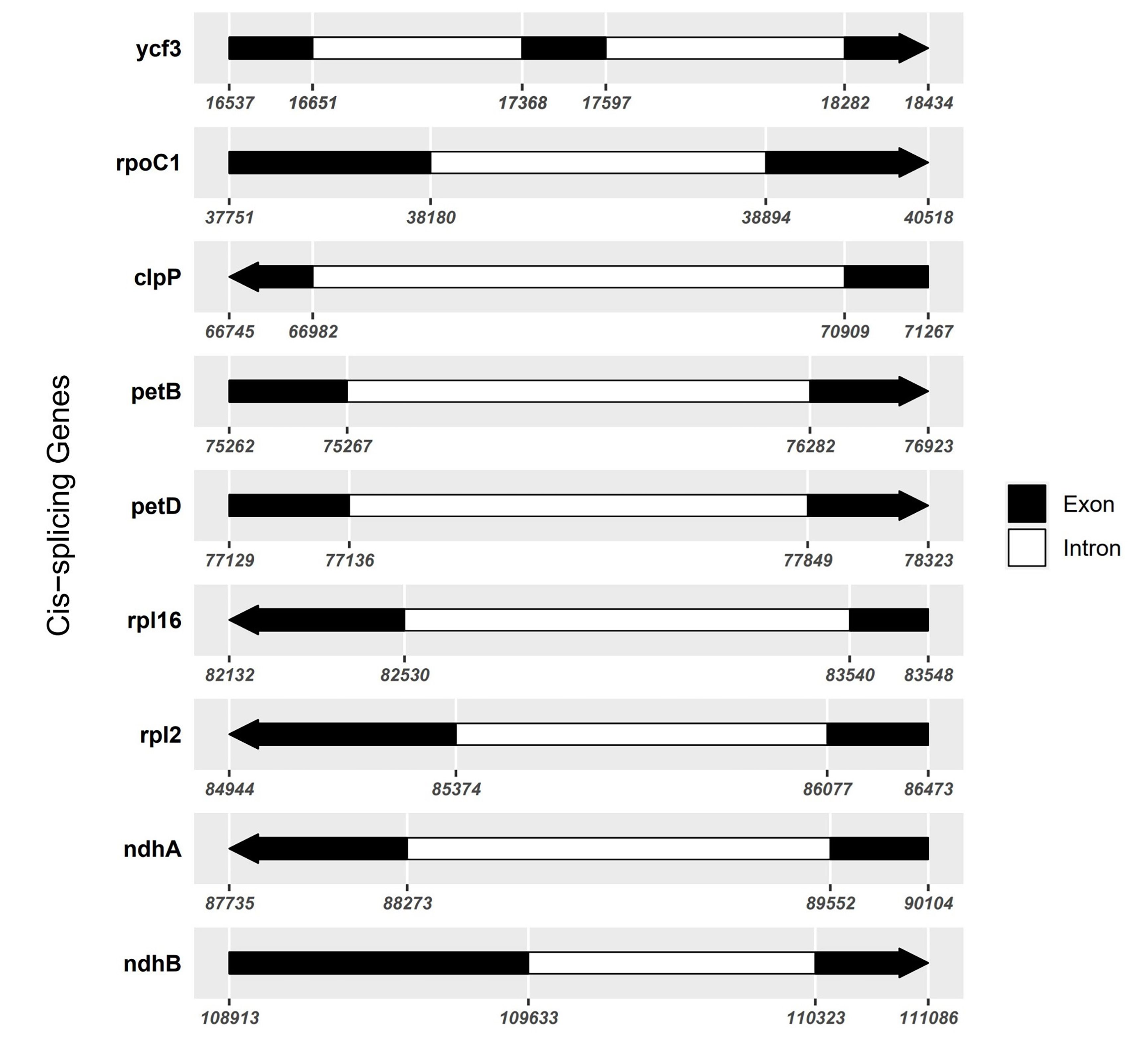


**Figure S3.** The map of the Repeat sequence analysis. For A map, horizontal coordinates indicate the type of SSR, vertical coordinates indicate the number of repetitive fragments, and the orange legend indicates monomeric SSRs, the blue legend indicates dimeric SSRs, the purple legend indicates trimeric SSRs, the green legend indicates tetrameric SSRs, and the grey legend indicates pentameric SSRs. For B map, horizontal coordinates indicate the type of repetitive sequences, vertical coordinates indicate the number of repetitive fragments, green legend indicates tandem repeats, blue legend indicates palindromic repeats, yellow legend indicates forward repeats, and red legend indicates reverse repeats.


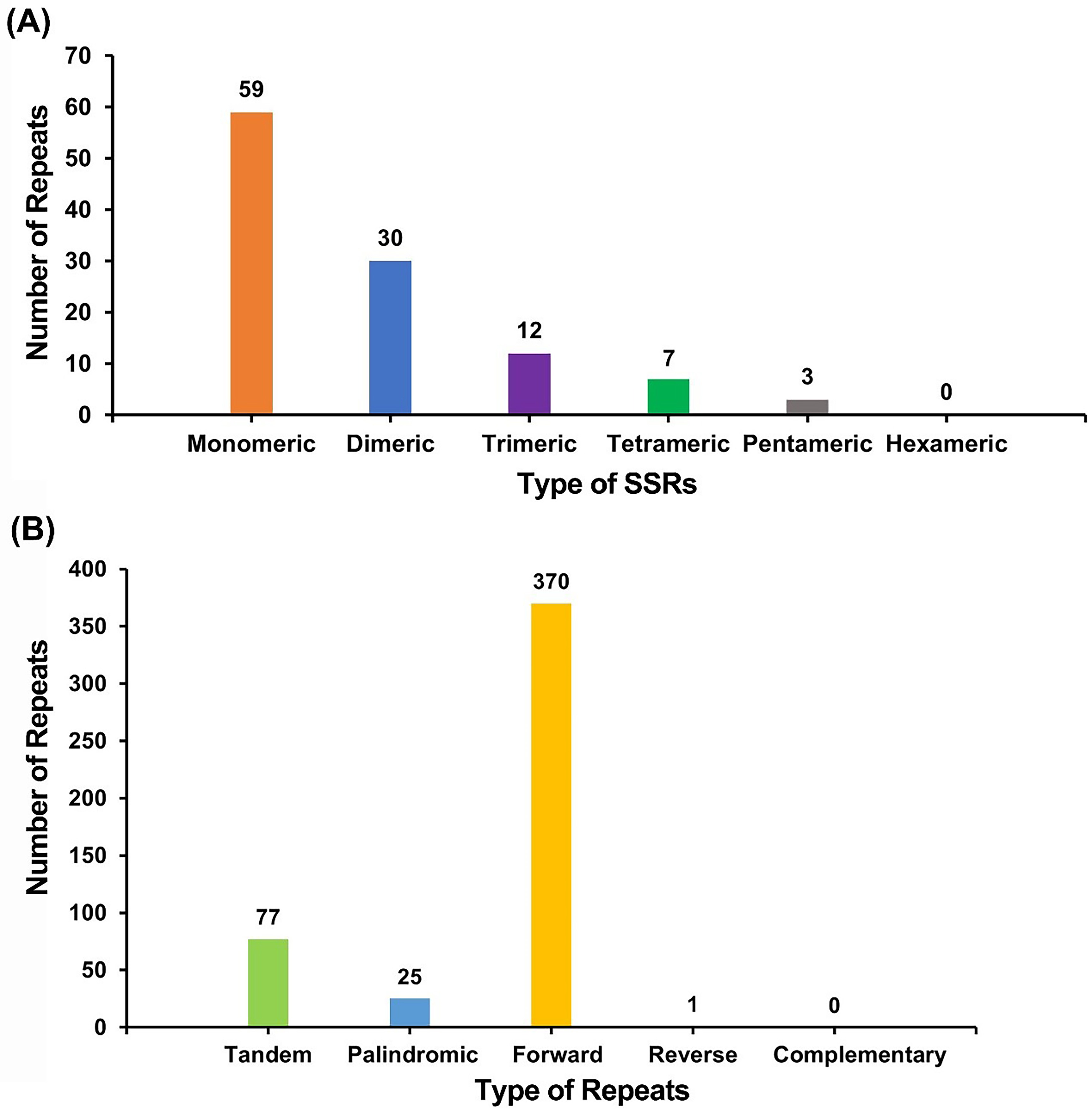


**Table S1.** The chloroplast-encoded genes of *Oxytropis kansuensis.*

Note: The number in parentheses represents the number of copies of the gene, e.g. (×2) means there are two copies.

| Group of genes | Name of genes |
| --- | --- |
| Subunits of NADH-dehydrogenase | *ndh*A, *ndh*B, *ndh*C, *ndh*D, *ndh*E, *ndh*F, *ndh*G, *ndh*H, *ndh*I, *ndh*J, *ndh*K |
| Subunits of photosystem Ⅰ | *psa*A, *psa*B, *psa*C, *psa*I, *psa*J, *psb*A, *psb*B, *psb*C, *psb*D, *psb*E, *psb*F, *psb*H |
| Subunits of photosystem Ⅱ | *psb*I, *psb*J, *psb*K, *psb*L, *psb*M, *psb*N, *psb*T, *psb*Z, *ycf*3 |
| Subunits of cytochrome b/f complex | *pet*A, *pet*B, *pet*D, *pet*G, *pet*L, *pet*N |
| Subunits of ATP synthase | *atp*A, *atp*B, *atp*E, *atp*F, *atp*H, *atp*I |
| Large subunit of rubisco | *rbc*L |
| Small subunit of ribosome | *rps*2, *rps*3, *rps*4, *rps*7, *rps*8, *rps*11, *rps*12, *rps*14, *rps*15, *rps*18, *rps*19 |
| Large subunit of ribosome | *rpl*2, *rpl*14, *rpl*16, *rpl*20, *rpl*23, *rpl*32, *rpl*33, *rpl*36 |
| DNA dependent RNA polymerase | *rpo*A, *rpo*B, *rpo*C1, *rpo*C2 |
| rRNA genes | *rrn*4.5S, *rrn*5S, *rrn*16S, *rrn*23S |
| tRNA genes | *trn*A-UGC, *trn*C-GCA, *trn*D-GUC, *trn*E-UUC, *trn*F-GAA, *trnf*M-CAU, *trn*G-GCC(×2), *trn*G-UCC, *trn*H-GUG, *trn*I-CAU, *trn*I-GAU, *trn*K-UUU, *trn*L-CAA, *trn*L-UAA, *trn*L-UAG, *trn*M-CAU, *trn*N-GUU, *trn*P-UGG, *trn*Q-UUG, *trn*R-ACG, *trn*R-UCU, *trn*S-GCU, *trn*S-GGA, *trn*S-UGA, *trn*T-GGU, *trn*T-UGU, *trn*V-GAC, *trn*V-UAC, *trn*W-CCA, *trn*Y-GUA |
| Maturase | *mat*K |
| c-type cytochrom synthesis gene | *ccs*A |
| Envelope membrane protein | *cem*A |
| Protease | *clp*P |
| Subunit of Acetyl-CoA-carboxylase | *acc*D |
| Genes of unknown functions Open Reading | *ycf*1, *ycf*2, *ycf*4 |
